# European brain research area: The operational level

**DOI:** 10.1192/j.eurpsy.2021.169

**Published:** 2021-08-13

**Authors:** K. Aarts, E. De Witte, S. Kramer, D. Iannone, F. Destrebecq, M. Di Luca

**Affiliations:** Eu-projects, European Brain Council, Bruxelles, Belgium

**Keywords:** Europe, Brain research, Coordination, Research community

## Abstract

Brain research in Europe is a rapidly evolving field, and increasingly at the forefront of science. Although considerable amounts of knowledge and innovative approaches have been generated, the translation into new health interventions is hindered by excessive fragmentation. Effective and efficient collaboration and cooperation among the various initiatives are often identified as a key success factor to achieve brain research full impact. EBRA fully responds to these needs by bringing together the various stakeholders and major brain research initiatives, at European level and beyond. EBRA creates the conditions for real and effective cross fertilisation, dialogue, building consensus and exploiting research potential. On the operational level, EBRA facilitates the emergence of research projects in specific areas in active clusters. A cluster is understood as a research community that can be directed towards basic research, clinical research and/or methodological approaches under a common topic and disease area within brain research. EBRA support clusters to: 1. Consolidate or expand further the research community expand their community, 2. Engage with policy makers and other relevant stakeholders, 3. Build consensus on various issues (research priorities, research roadmap, data sharing, etc.), 4. Promote links with existing research infrastructures, 5. Increase the visibility of the research community through communication and dissemination activities, 6. Coordinate the development of position/consensus papers, white papers, guidelines, meeting reports and/or other cluster outcomes. EBRA currently has 6 existing clusters: EPICLUSTER, Prevention of Severe Mental Disorders (PSMD)-cluster, TRISOMY21-cluster, BRAINFOOD-cluster., PREMOS-cluster and ECIB-cluster.

**Disclosure:**

No significant relationships.

